# Infrapatellar versus suprapatellar approach for intramedullary nailing of the tibia: a systematic review and meta-analysis

**DOI:** 10.1186/s13018-021-02249-0

**Published:** 2021-01-28

**Authors:** Nikhil Ponugoti, Branavan Rudran, Amr Selim, Sam Nahas, Henry Magill

**Affiliations:** 1 Basingstoke and North Hampshire Hospital, Basingstoke, UK; 2 Chelsea and Westminster Hospital, London, UK; 3 Royal Cornwall Hospital, Truro, UK; 4 Hillingdon Hospital, London, UK

**Keywords:** Tibia fracture, Fixation, Suprapatellar, Infrapatellar

## Abstract

**Background:**

Intramedullary nailing (IMN) is a conventional technique for the treatment of tibial shaft fractures. It has been suggested that the suprapatellar (SP) approach holds advantages over the traditional infrapatellar (IP) approach. Current literature lacks adequate data to provide robust clinical recommendations. This meta-analysis aims to determine the efficacy of infrapatellar versus suprapatellar techniques for IMN.

**Methods:**

An up-to-date literature search of the Embase, Medline, and registry platform databases was performed. The search was conducted using a predesigned search strategy and all eligible literature was critically appraised for methodological quality via the Cochrane’s collaboration tool. Fluoroscopy time, operative time, pain score, knee function, deep infection, non-union and secondary operation rates were all considered.

**Conclusion:**

A total of twelve studies were included in the meta-analysis. The results of this analysis show that suprapatellar nailing is associated with reduced post-operative pain scores and improved functional outcomes. The data suggest no significant difference in terms of operative times, fluoroscopy times, rates of deep infection, non-union or secondary procedures when compared to infra-patellar techniques. Further studies are required to confirm these findings and assess long-term results.

## Background

Tibial shaft fractures represent the most common diaphyseal fractures in adults and account for approximately 2% of all fractures [1]. There are various treatment modalities for operatively managing these injuries including open reduction and internal fixation, external fixation and intramedullary nailing (IMN). The current standard of care for surgically managed tibial shaft fractures is IMN. IMN allows for minimal soft tissue disruption, conservation of the periosteal blood supply, early mobilisation and weight-bearing. Higher union rates and fewer wound complications have also been reported [2, 3].

Intramedullary nails have traditionally been inserted through an infrapatellar (IP) approach that is typically performed with the knee in flexion. The nail is inserted either through a trans- or parapatellar technique. The most commonly cited complication of the IP technique is post-operative anterior knee pain, with an incidence between 10 and 80% [4–7]. Additionally, the IP approach can be technically challenging due to proximal fragment displacement caused by the extensor complex on the flexed knee.

More recently, the suprapatellar (SP) method of nail insertion has been introduced and fast becoming a familiar alternative [8]. The SP approach involves splitting the quadriceps tendon; a flexible cannula is then placed into the suprapatellar space and subsequently into the retro-patellar space. This allows for the insertion of a standard nail system using a simple extension to the jig. The technique aims to addres**s** the disadvantage of proximal fragment migration by maintaining the knee in a semi-extended throughout the procedure [9]. Franke et al. also suggests the approach aids fracture reduction and simplifies intra-operative imaging [9].

A recent meta-analysis in 2018 demonstrates the SP approach holds significant advantage over IP intramedullary techniques; however, the authors acknowledge the low quality of the available evidence and requirement for further high-quality randomised controlled trials (RCTs) [10]. A further meta-analysis in the same year suggested superiority of the SP approach with significantly shorter fluoroscopy time, a lower VAS pain score and no increased risk of post-operative complications. Wang et al. recognise the low sample sizes and significant heterogeneity in the data and have suggested that results be treated with caution [11].

The current literature therefore suggests an advantage of SP over IP intramedullary nailing; however, significant limitations in the data make robust conclusions challenging. In order to comprehensively scrutinise the literature and provide stronger clinical recommendations, we have conducted the most up-to-date meta-analysis to evaluate the outcomes of suprapatellar versus infrapatellar nailing techniques.

## Methods

### Literature search

Using Preferred Reporting Items for Systematic reviews and Meta-analyses (PRISMA) [12] and the Cochrane Handbook for Systematic Review of Intervention, Version 5.1.0 [13] a systematic review and quantitative analysis were performed. We have searched the Medline and Embase databases up to May 2020. The search was performed on the following 3 areas: ‘Tibial fractures’ [Mesh] or ‘Tibia’ [Mesh], ‘Fracture Fixation, Intramedullary’ [Mesh] and ‘*Patella’ OR ‘*Knee Cap’. We have initially narrowed down our search on ‘Tibia fractures’ to ‘Patella’ (knee cap) and then looked at fracture fixation methods.

### Searching other resources

An additional search was also performed for previously published, planned and on-going trials by identifying references in ClinicalTrials.gov (http://clinicaltrials.gov/) and the World Health Organisation (WHO) International Clinical Trials Registry Platform search portal (http://apps.who.int/trialsearch/).

### Inclusion and exclusion criteria

All search terms, titles, abstracts and full text of articles that were deemed suitable for abstract were reviewed by two of the study’s authors (NP and BR). Any disagreement regarding the choice of included studies was resolved by consensus amongst all four co-authors.

#### Inclusion criteria

1. Level I, level II, level III (prospective and retrospective comparative studies) evidence

2. Studies comparing IP to SP approaches in treating tibia fractures

3. Subjects above 17 years of age

4. Human research

5. English language only

#### Exclusion criteria

1. Cadaveric or animal studies

2. Studies primarily evaluating biomechanical properties of either approaches

3. Abstracts, case reports, case series, letters and conference articles

4. Studies with insufficient data

### Outcome measures

The primary outcome measures of interest for this review were as follows:

1. Fluoroscopy time (minutes)

2. Operating time (minutes)

3. Visual Analogue Scale for pain

4. Functional scores

5. Deep infection rates

6. Non-union rates

7. Secondary operation rates

### Data extraction

Primary outcome data from the selected studies were entered into Microsoft Excel (2013). All data extraction was performed by two independent co-authors where no discrepancies existed. Study characteristics were recorded in Table 1. Data Synthesis and Statistical Analysis Review Manager 5.3 was used for all data synthesis and subsequent analysis. All continuous outcome data were evaluated and the mean difference between the IP and SP groups was determined. All discrete data were assessed by evaluating the risk ratio between the IP and SP groups. *P* values were calculated and recorded for each primary outcome measure.

**Table 1:** Characteristics of the included studies

Author	Country	Design	Level of evidence	No. of patients (IP/SP)	Age (mean, year)	Gender (M:F) (IP/SP)	Follow-up (mean, month)	Loss of follow-up	AO/OTA classification	No. of open fractures
Avilucea 2016 [14]	USA	Retrospective cohort	III	134/132	35.4/33.6	76:56/88:44	NR	NR	43 A, C1, C2	31/28
Chan 2016 [15]	USA	RCT	II	18/23	43/40	10:4/6:5	14.4/16.7	12/4	42 A, B, C	1/2
Courtney 2015 [16]	USA	Retrospective cohort	III	24/21	37.6/38.5	11:13/15:6	25.2/11.8	NR	42 A, B, C	9
Cui 2019 [17]	China	Retrospective cohort	III	26/24	44.81/41.71	3:23/16:8	23.08/23.92	None	42 A, B, C	0
Isaac 2019 [18]	USA	Retrospective cohort	III	171/91	40.1/43.9	109:65/69:22	50.4/43.2	NR	NR	NR
Jones 2014 [19]	UK	Retrospective cohort	III	38/36	39/40	22:16/26:10	28/22	6/9	42 A, B, C	3/7
LI sheng-long 2017 [20]	China	Retrospective cohort	III	30/38	43.20/40.24	27:3/33:5	09-Sep	NR	41 A, 42 A, B, C, 43 A	NR
Macdonald 2019 [21]	UK	RCT	II	42/52	37.6/42.4	26:16/33:20	NR	17/15	42 A, B, C	8/7
Marececk 2017 [22]	USA	Retrospective cohort	III	142/147	32.7/39.7	114:28/131:16	10.9/9.4	NR	42 A, B, C	142/147
Ozcan 2018 [23]	Germany	Retrospective cohort	III	37/21	33.8/31	26:11/19:2	35.4/16	NR	42 A, B, C	0
Sun 2016 [7]	China	RCT	II	81/81	46.79/47.47	65:16/66:15	24/24	6/7	42 A, B, C	NR
Williamson 2018 [24]	UK	Retrospective cohort	III	37/53	NR	NR	NR	NR	NR	NR

A ‘random-effects’ model was only applied if high levels of heterogeneity existed between the studies for each outcome measure. Heterogeneity was determined with *I*^2^ that indicates the percentage of variance attributable to study heterogeneity. Zero to 25% indicates low heterogeneity, 25 to 75% indicates moderate heterogeneity, and > 75% suggests high heterogeneity. Final results for each outcome measure were displayed in a forest plot with the associated confidence intervals (CI).

### Methodological quality assessment

Two co-authors (NP and BR) independently evaluated the quality and the associated risk of bias of all the RCT’s according to Cochrane Handbook for Systematic reviews and interventions [13]. In order to assess the quality of randomised controlled trials, the following parameters used evaluated: (1) randomisation, (2) concealment of allocation, (3) blinding of participants in the study, (4) blinding of outcome assessment, (5) incomplete outcome data, (6) selective outcome reporting and (7) other bias. For non-randomised studies, a star rating system namely Newcastle-Ottawa scale has been used [25]. This scale grades the study from 0 to 9, where six or more is considered as a high-quality study. Any disagreement regarding the level of bias was resolved by consensus amongst all four co-authors.

## Results

### Literature search results

**Fig. 1:**
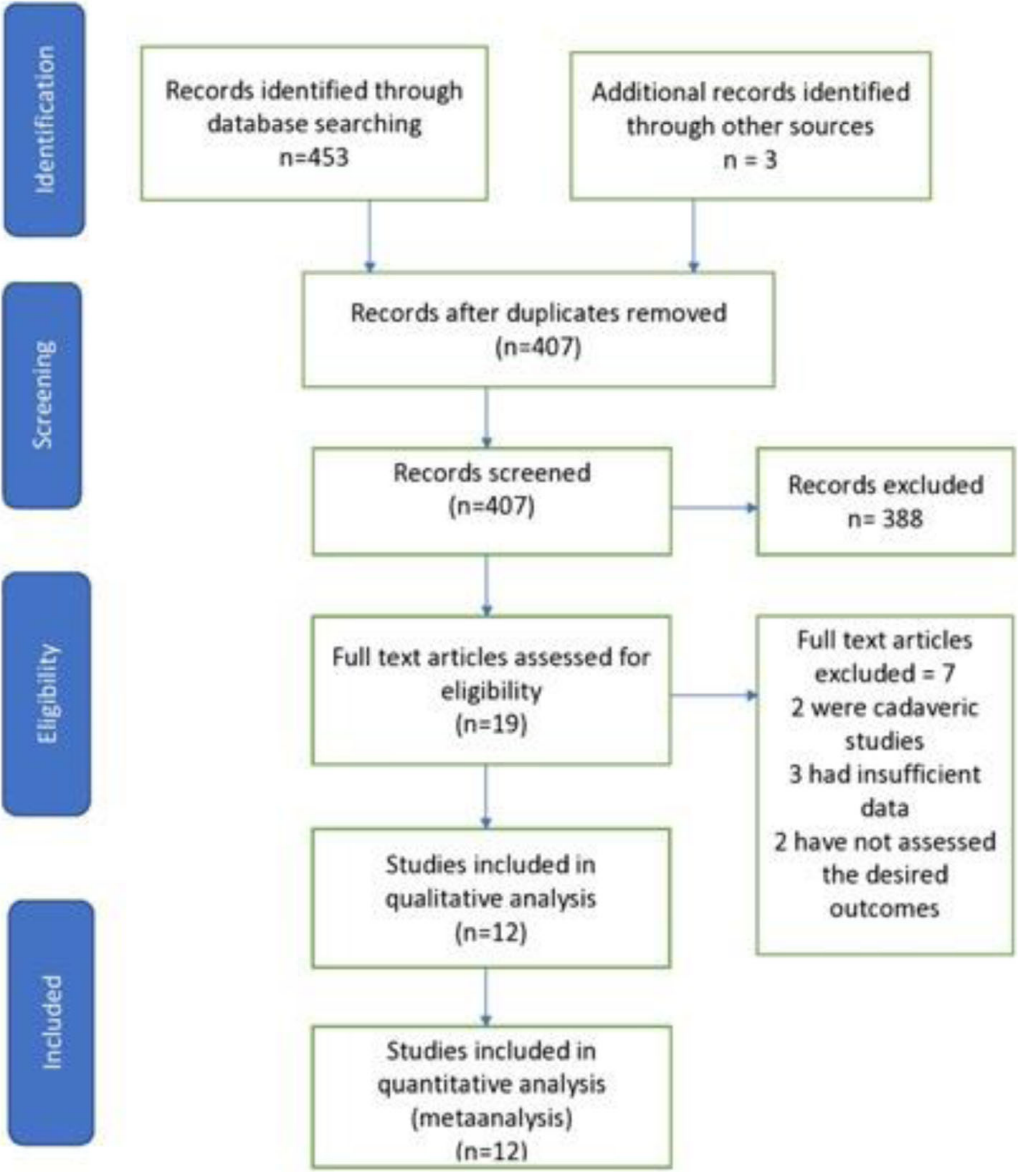
The Preferred Reporting Items for Systematic reviews and Meta-analysis

The initial search of the databases yielded 453 studies from Medline and Embase. Finally, three RCTs and 9 retrospective cohort studies were deemed eligible for the meta-analysis. The PRISMA flow diagram for this search is shown in Fig. 1 demonstrates our search strategy.

### Quality assessment

**Fig. 2:**
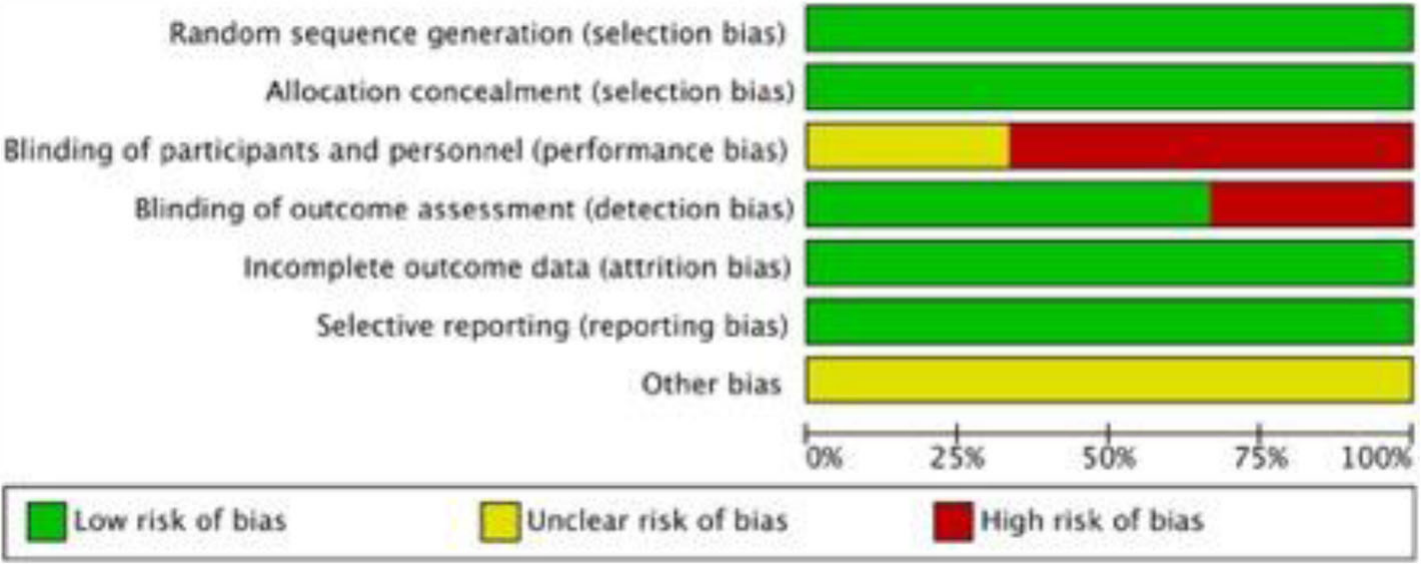
The risk of bias graph of the included studies. The colour represents the quality in the each of the domains (high = high risk, unclear = uncertain and low = low risk)

**Fig. 3:**
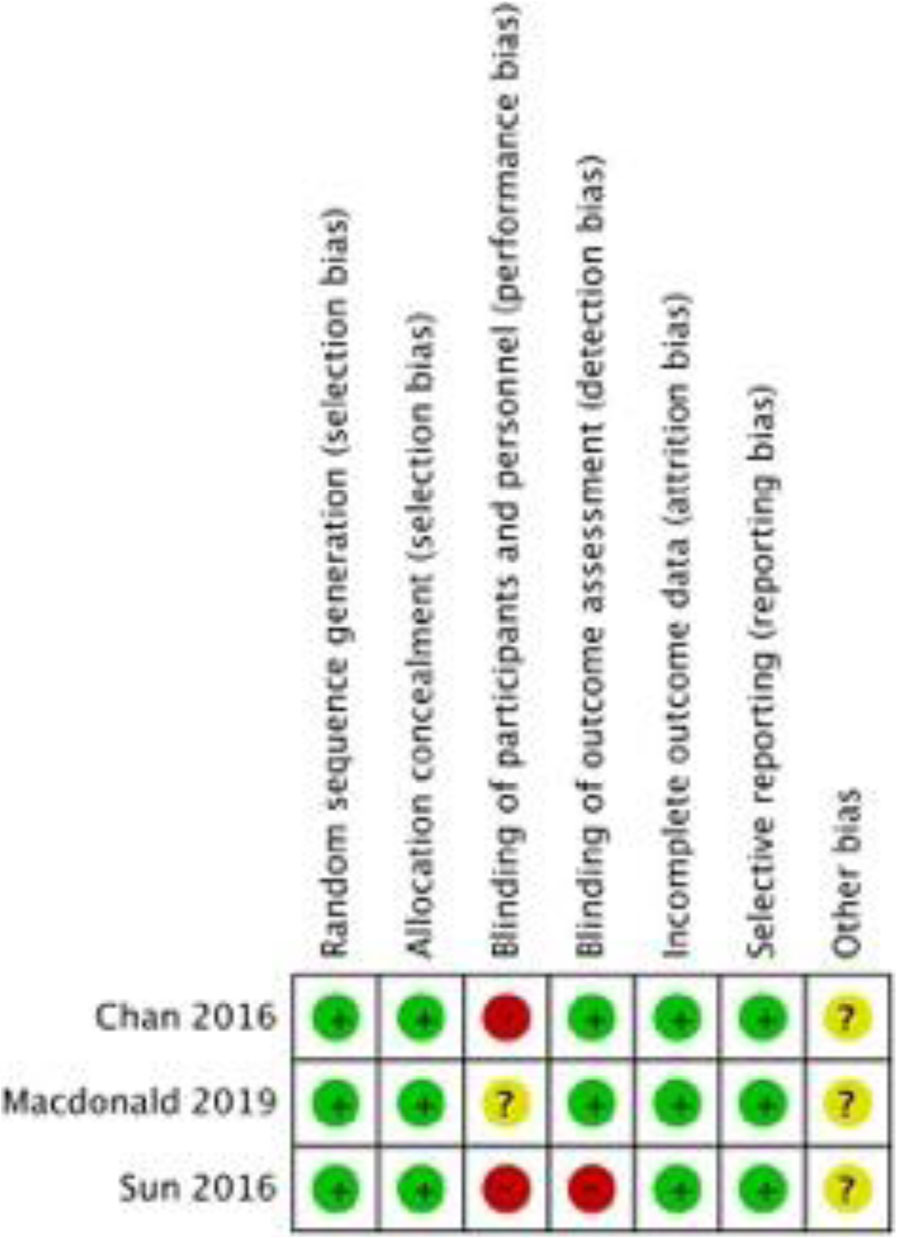
The risk of bias for each of the included studies. The colour represents the quality in each of the domains (red = high risk, yellow = uncertain and green = low risk)

The majority of the RCTs have low risk of bias in terms of randomisation, allocation concealment, incomplete outcome data and selective outcome reporting, whereas some studies demonstrated high levels of bias in terms of blinding of participants and outcome assessment [7, 15, 21]. The risk of bias graph and summary are displayed in Figs. 2 and 3.

All non-randomised studies were assessed against the Newcastle-Ottawa score for comparative studies with a subjective score out of 9. A table illustrating the scores is shown in Table 2.

**Table 2:** The methodological index for non-randomised studies (Newcastle-Ottawa scale)

	Selection	Comparability	Exposure	Total score
Study				
Avilucea 2016 [14]	3	2	3	8
Courtney 2015 [16]	3	2	2	7
Cui 2019 [17]	3	2	3	8
Isaac 2019 [18]	3	2	3	8
Jones 2014 [19]	3	2	3	8
Li sheng-long 2017 [20]	3	2	2	7
Marecek 2017 [22]	3	1	3	7
Ozcan 2018 [23]	3	2	1	6
Williamson 2019 [24]	3	2	3	8

### Characteristics of studies included

The details of the 3 RCTs and 9 comparative studies included in the systematic review are summarised in Table 1. All included studies were published between 2014 and 2019. In total, 12 studies included 1499 patients. A total of 780 were managed operatively via the infrapatellar approach and 719 via the suprapatellar approach. The median follow up time periods for each study ranged from 9 months to 50.4 months. Post-operative knee function was assessed with the Lysholm knee score in four studies [7, 15, 21, 23]. the Hospital for Special Knee Surgery Score (HSS) in 2 studies [17, 20], Kujala Knee Score in 2 studies [19, 23] and Oxford Knee Score (OKS) in one study [16]. Pain scores assessed by Sun et al. and Chan et al. with the VAS pain scoring system, where Isaac et al. used NRS pain scoring system [7, 15, 18]. Even though NRS pain score is moderately higher than the VAS score, both are highly correlated pain scores [26].

### Outcome 1: Fluoroscopy time

**Fig. 4:**
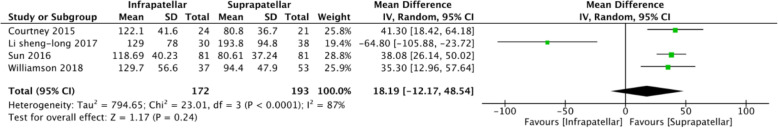
Forest plots of the comparison of fluoroscopy time between the two approaches. Abbreviations: CI, confidence interval; IV, independent variable; M-H:, Mantel-Haenszel

The fluoroscopy time was reported in 4 studies (*n* = 193) with high level of heterogeneity (*I*^2^ = 87%) [7, 11, 16, 20, 24]. Comparison of SP to IP in terms of fluoroscopy time with random effect analysis was not significant (Fig. 4).

### Outcome 2: Operation time (minutes)

**Fig. 5:**
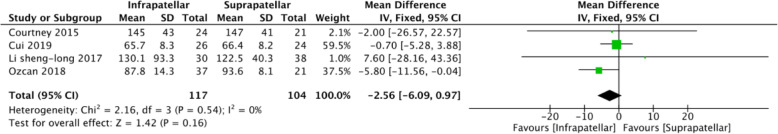
Forest plots of the comparison of operation time. Abbreviations: CI, confidence interval; IV, independent variable; M-H, Mantel-Haenszel

The operation time was reported in 4 studies (*n* = 104) with a lowest level of heterogeneity (*I*^2^ = 0%) [16, 17, 20, 23]. The difference between SP and IP group is not statistically significant (Fig. 5).

### Outcome 3: Pain scores

**Fig. 6:**
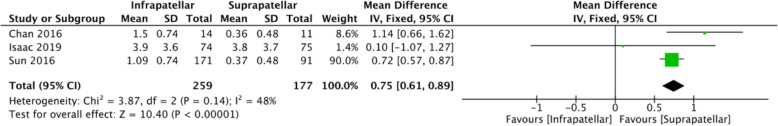
Forest plots of the comparison of pain scores. Abbreviations: CI, confidence interval; IV, independent variable; M-H, Mantel-Haenszel

Pain scores were reported in 3 studies (*n* = 177) with a moderate level of heterogeneity (*I*^2^ = 48%) [7, 15, 18]. The comparative analysis suggests that suprapatellar group had better pain scores when compared to the infrapatellar group (Fig. 6).

### Outcome 4: Knee functional scores

**Fig. 7:**
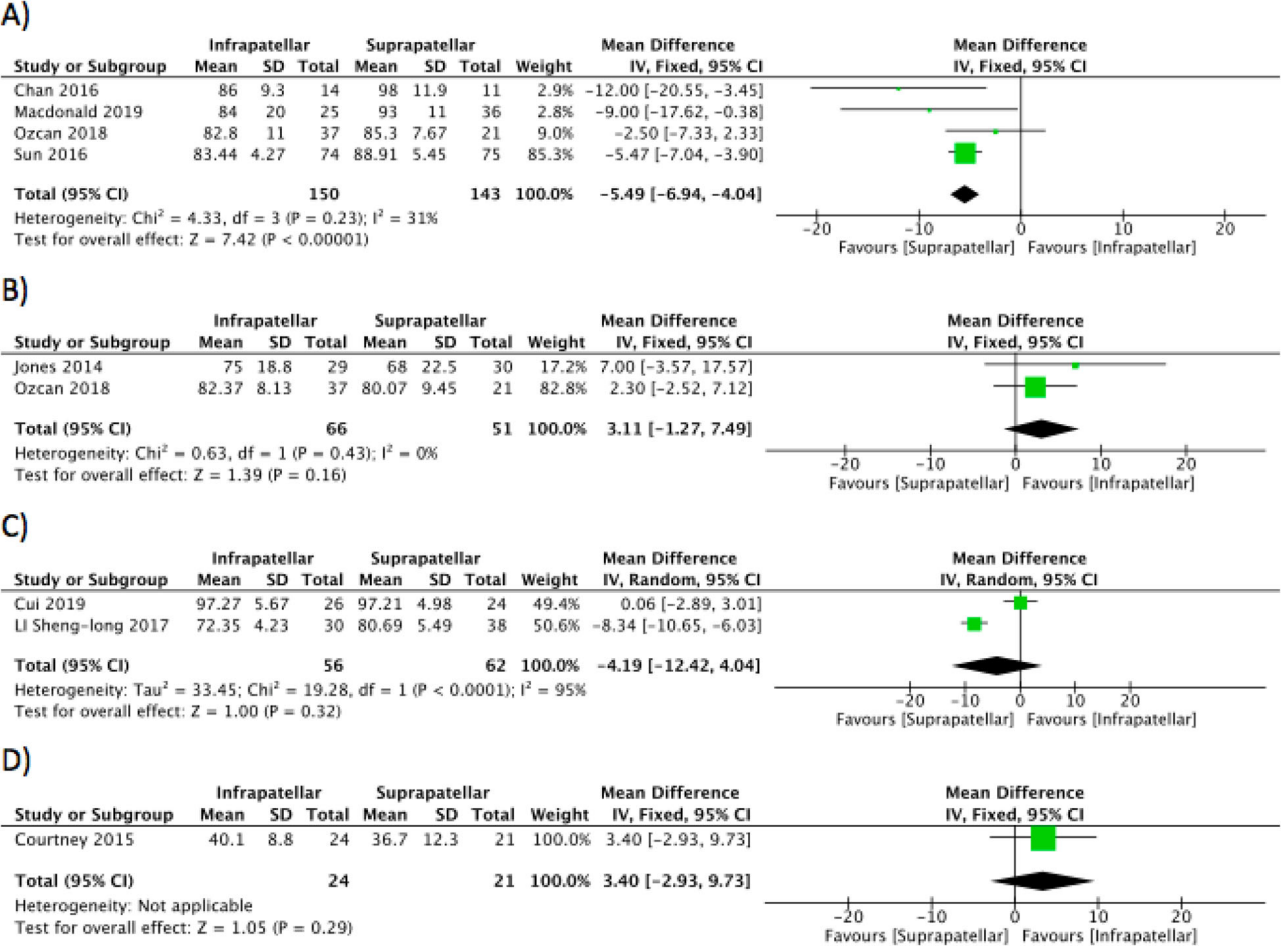
Forest plots of the comparison of knee functional scores. (**a**) Lysholm scores, (**b**) HSS scores, (**c**) Kujala scores and (**d**) OKS respectively. Abbreviations: CI, confidence interval; IV, independent variable; M-H, Mantel-Haenszel

Post-operative knee function was assessed with the Lysholm knee score in four out of eight studies [7, 15, 21, 23], and the results show that there is moderate heterogeneity with better functional outcomes in SP group at 12 months. The studies with outcomes of HSS score, Kujala score, and Oxford knee score were unable to show any superiority of SP group (Fig. 7).

### Outcome 5: Deep infection

**Fig. 8:**
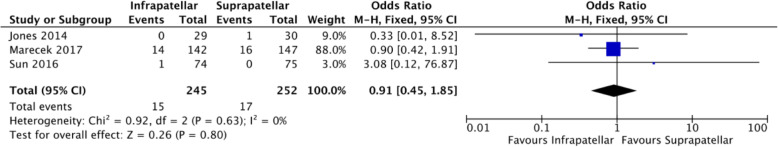
Forest plots of the comparison of deep infection. Abbreviations: CI, confidence interval; IV, independent variable; M-H, Mantel-Haenszel

The deep infection was reported in 3 studies (*n* = 252) with no heterogeneity (*I*^2^ = 0%) [7, 19, 22]. There was no significant difference between SP vs. IP (Fig. 8).

### Outcome 6: Non-union rate

**Fig. 9:**
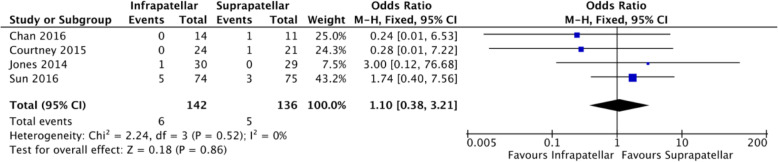
Forest plots of the comparison of non-union rate. Abbreviations: CI, confidence interval; IV, independent variable; M-H, Mantel-Haenszel

The non-union rate was reported in 4 studies (*n* = 136) with no heterogeneity (*I*^2^ = 0%) [7, 15, 16, 19]. The difference between SP and IP was not statistically significant (Fig. 9).

### Outcome 7: Secondary operation

**Fig. 10:**
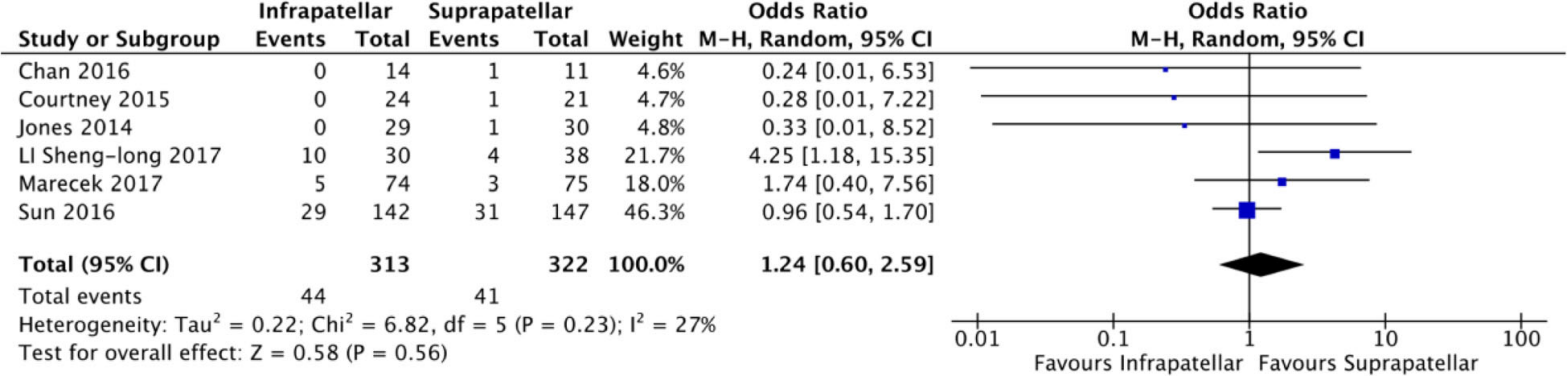
Forest plots of the comparison of secondary operation. Abbreviations: CI, confidence interval; IV, independent variable; M-H, Mantel-Haenszel

Six studies compared secondary operation rates (*n* = 322) with moderate heterogeneity (*I*^2^ = 27%) [7, 15, 16, 19, 20, 22]. The rate of secondary operation did not differ when compared to suprapatellar and infrapatellar groups (Fig. 10).

### Sensitivity analysis

We performed a sensitivity analysis for all statistically significant results; the random effect model was used for the comparisons with high heterogeneity whereas fixed effect model was used for comparisons with low to moderate heterogeneity. Both fixed and random effects models were applied to pain and functional scores; the results remained significant.

## Discussion

This is the most-up-date and extensive meta-analysis to compare the suprapatellar to the infrapatellar approach for tibial IMN. The data in our study indicate that the suprapatellar approach is associated with reduced post-operative pain scores when compared to infrapatellar approach. Authors have hypothesised that post-operative knee pain is due to patellar tendon splitting, proximal nail protrusion, intra-articular structural damage and involvement of the infrapatellar nerve; the suprapatellar approach aims to avoid this [6, 10]. However, it is difficult to conclude superiority of suprapatellar approach in terms of less post-operative pain as only 3 out of 12 studies used similar pain scoring systems [7, 15, 18]. Few other studies did mention about knee pain, but, unfortunately, they could not be included in our analysis due to insufficient reported data [17, 21, 23]. Macdonald et al. [21] suggest a lesser anterior knee pain in the SP group at 4 months post-operatively whereas there was no significant difference between IP and SP groups according to Ozcan et al. and Cui et al. [17, 23].

With regards to functional outcome, articles using HSS score or Kujala score or Oxford knee score have shown no difference in between the approaches, but 4 of the studies [7, 15, 21, 23] which used Lyslohm knee score have demonstrated significantly better functional outcome in the suprapatellar group at 12 months. Undoubtedly improved post-operative pain positively impacts the ability to rehabilitate; the improved functional scores observed with the suprapatellar group are therefore likely to be attributable to the lower pain scores recorded.

The data from our meta-analysis have shown no significant difference in terms of operative and fluoroscopy times. Interestingly, a recent meta-analysis by Wang et al. in 2018 demonstrated shorter fluoroscopy time despite no difference in overall operative time. The authors suggest this finding was due to the simplicity of fluoroscopy positioning whilst the knee was in a semi-flexed position [11]. Our meta-analysis does, however, include more, recently published high-quality studies [18, 20–24]. This data, when pooled, clearly show no difference in fluoroscopy time. This result is consistent with the finding that the overall operative time remains unaffected.

The data have also demonstrated no significant difference in terms of the rates of deep infection, non-union or secondary procedures. This is in keeping with previously published data [11].

Malalignment is one of the noted complications to intramedullary nailing of the tibia. Courtney et al. mentioned that post-operative sagittal plane malalignment is lesser with the SP group (2.90°) compared to the IP group (4.58°) in tibial shaft fractures [16]. Avilucea et al. and Lu et al. have suggested lesser degree of malalignment with the SP group in distal tibia fractures [14, 27]. This has also been observed in extra-articular proximal tibia fractures as per Kulkarni et al. [28]. Hyperflexion of the knee during infrapatellar nailing may preclude the ideal entry point and also could create difficulty in maintaining alignment especially in proximal and distal tibia fractures [29, 30]. Further high-quality RCTs are required to establish robust conclusions.

Some limitations of the present study should be highlighted; only twelve studies with a total of 1499 patients were included in the analysis. Nine of these studies were retrospective cohort studies that may lower the quality of the data included. All included studies are at risk of bias, largely because of inherent impracticality of blinding both participants and surgeons. Additionally, other important parameters such as union time, range of motion, ease of surgery, duration and maintenance of reduction were not compared across the included studies.

Heterogeneity was noted to be moderate to high in those forest plots, namely, pain and functional scores. The duration of follow-up was variable in the included studies; this may lead to higher levels of heterogeneity when assessing pain and functional scores. The minimum follow-up in this meta-analysis was 12 months for both pain and functional assessment except for one study [20], which has collected functional outcomes at 9 months. Unfortunately, we are unable to provide a mean follow-up time and progression of scores over time, as only a few studies published a specific timeline when assessing these outcomes.

Additionally, a major concern of the suprapatellar approach is the potential for chondral damage of the patellofemoral joint (PFJ). A number of small studies have assessed the chondral surfaces following SPN using arthroscopy, MRI imaging and clinical examination [15, 31]. These studies appeared to have no consistent findings where overall subject numbers and follow-up time were low. Chan et al. suggests that PFJ injury could be avoided with diligent nail placement but recommended that further RCTs with larger subject numbers and long-term follow-up was required [15].

## Conclusion

The results of this meta-analysis show that suprapatellar nailing is associated with reduced post-operative pain scores and improved functional outcomes. The data have suggested no significant difference in terms of operative times, fluoroscopy times, rates of deep infection, non-union or secondary procedures when compared to infra-patellar techniques. Further studies are required to confirm these findings and assess long-term results.

## Data Availability

Yes

## Abbreviations

IMN: Intramedullary nail
SP: Supra-patellar
IP: Infrapatellar
RCT: Randomised controlled trial
PRISMA: Preferred reporting items for systematic review and meta-analysis
HSS: Hospital for special knee surgery score
OKS: Oxford knee score
NRS: Numerical rating system for pain
PFJ: Patellofemoral joint
